# Frequency of Granulomatous Invasive Fungal Sinusitis in Patients with Clinical Suspicion of Chronic Fungal Rhinosinusitis

**DOI:** 10.7759/cureus.4757

**Published:** 2019-05-25

**Authors:** Muhammad Shahid Sharif, Salman Ali, Hasan Nisar

**Affiliations:** 1 Otolaryngology, Nishtar Hospital, Multan, PAK

**Keywords:** chronic fungal rhinosinusitis, frequency, granulomatous invasive fungal sinusitis, granulomatous fungal invasion, nasal biopsy, pakistan, chronic fungal rhinosinusitis

## Abstract

Introduction

One of the common causes of chronic sinusitis is a fungal infection, and there are various types of fungal rhinosinusitis (FRS). Missed diagnosis of occasional granulomatous invasion of fungal sinusitis can lead to involvement in the central nervous system. The aim of this study is to determine the frequency of granulomatous invasive fungal sinusitis (GIFS) in patients with clinical suspicion of chronic FRS.

Methods

We conducted a descriptive cross-sectional study in the Department of Ear, Nose and Throat (ENT), Nishtar Hospital, Multan from January 1, 2017 to July 1, 2018. Eighty-one patients with chronic FRS participated in the study. After informed consent, nasal tissue was biopsied for granulomatous fungal invasion.

Results

The frequency of GIFS was 29.6% (n=24) in this study. The significant risk factors included duration of chronic FRS for more than 12 weeks, history of diabetes mellitus, and living status as rural.

Conclusion

GIFS is a common complication in patients with clinical suspicion of chronic FRS. Nasal biopsy should be a common practice among patients of chronic FRS who have a long duration of disease and a history of diabetes mellitus.

## Introduction

One of the most common problems addressed by an Ear, Nose and Throat (ENT) specialist is chronic rhinosinusitis. It is classically characterized by nasal blockage, anterior and/or posterior nasal discharge, cephalalgia and facial pain, and anomalies in smelling [1]. Fungal sinusitis has been imputed for triggering most conditions of chronic rhinosinusitis. When fungal spores are inhaled, they can start colonization and cause intrusive or nonintrusive types of rhinosinusitis, depending on the host’s immune system. Consequently, fungal rhinosinusitis (FRS) is divided into two immense branches, invasive and noninvasive, which can be distinguished by their invasion capability on the mucosal membrane [2]. Critically invasive, inveterately invasive, and granulomatous are the three forms of intrusive FRS [3]. Fungi-linked eosinophilic rhinosinusitis comprising adverse FRS and fungal ball are two noninvasive types of FRS [4-5].

Fungal infections differ according to specific regions. Granulomatous fungal sinusitis, characteristically of gradual onset, has been principally designated in India, Pakistan, and Sudan [6-7]. However, whether this affliction is regional or related to some specific race is unknown. A report on the prevalence of FRS by Challa et al. [8] revealed an FRS incidence of 45.7%, of which granulomatous intrusive occurred in 30%, allergic in 23.8%, chronic noninvasive in 1.6%, chronic intrusive in 15.87%, and acute fulminant in 28.5%. A wrongly diagnosed or missed case of occasional granulomatous invasive fungal sinusitis (GIFS) leads to its involvement into the central nervous system or the orbit which can intensify the disease.

Lack of existing data and insufficient knowledge on this disease and its complications have resulted in a major gap in medical knowledge regarding this disease. So, the main objective of this research is to evaluate GIFS in people with FRS. Based on our findings, we could organize public awareness events on local and national levels to incite target patients to see a physician in a timely manner should any symptoms of this disease occur, and appropriate therapy could be given to them to minimize the illness.

## Materials and methods

This study is a descriptive cross-sectional study conducted at the Department of ENT at Nishtar Hospital, Multan from January 1, 2017 to July 1, 2018. Ethical approval for the study was obtained from our institutional ethics committee. We used non-probability consecutive sampling technique to collect our data. We identified 109 patients of chronic FRS during the study period. After losing patients due to lack of consent and follow-up, a total of 81 patients completed the study. All patients were aged 18 years or older. Patients with a history of previous trauma to the nose or face or previously diagnosed cases of fungal sinusitis, any patients who previously had undergone sinonasal intervention or surgery, and patients unwilling to take part in the study were excluded.

Surgical resection or biopsies of the nasal masses were taken by a consultant surgeon with experience of at least five years of fellowship. The biopsied tissue was sent for histopathological examination to evaluate the presence or absence of GIFS. All the data including demographic variables and frequency of GIFS were collected via a pre-formed proforma by the researcher himself.

Data thus collected were subjected to statistical analysis using the IBM SPSS Statistics for Windows, Version 23.0 (IBM Corp., Armonk, NY). Mean and standard deviation were calculated for quantitative variables like the duration of the disease and age of the patients while frequency and percentage were calculated for categorical variables such as gender and frequency of GIFS. The chi-square test was used to check the association between the variables, and a P≤0.05 was considered statistically significant.

## Results

Of the 81 patients with chronic FRS, GIFS was diagnosed in 24 (29.6%). There were 16 (66.7%) male patients and eight (33.3%) female patients in the GIFS groups. Their mean age and duration of disease was 33 ± 2 years and 10 ± 3 weeks, respectively.

We compared the characteristics of the patients categorized according to their diagnosis of GIFS. GIFS was more frequent in men and younger patients. The incidence of GIFS showed a statistically significant correlation with a longer duration of FRS, comorbidity of diabetes, and living status (Table 1).

**Table 1:** Patient characteristics and incidence of granulomatous invasive fungal sinusitis

Patients characteristics	Granulomatous invasive fungal sinusitis		P value
	Present (n=24; 29.6%)	Not present (n=57; 70.3%)	
Gender			
Male	16 (66.7%)	35 (61.4%)	0.654
Female	8 (33.3%)	22 (38.6%)	
Age in years			
20-35 years	16 (66.7%)	32 (56.1%)	0.379
36-50 years	8 (33.3%)	25 (43.9%)	
Duration of disease in weeks			
7-12 weeks	8 (33.3%)	49 (86%)	0.046
More than 12 weeks	16 (66.7%)	8 (14%)	
Status of diabetes			
Diabetes present	8 (33.3%)	5 (8.8%)	0.016
Unknown/no diabetes	16 (66.7%)	52 (91.2%)	
Residential status			
Rural	16 (66.7%)	25 (43.9%)	0.061
Urban	8 (33.3%)	32 (56.1%)	

## Discussion

Fungi are present in all kinds of environments. Depending on the environmental conditions and the host, the fungi can take the form of an unharmful saprophyte or can result in an invasive fungal infection. The increase in the frequency of fungal infections has led to a significant number of morbidities as well as mortalities over the past decade and can be attributed to the extensive use of antibiotics, chemotherapy, immunosuppressive therapy, intensive care interventions, and an increased number of immunodeficiency diseases [9].

Fungi can colonize the paranasal sinuses and nose in both diseased and healthy populations and are often found on clinical examination. The prevalence of FRS is increasing daily, and FRS affects patient quality of life and impairs daily functioning. FRS presents itself in five clinical forms [10]. Each form of FRS presents with different criteria for diagnosis, treatment modalities, and prognosis of the disease. There are three invasive forms: acute fulminant, granulomatous, and chronic invasive FRS. Non-invasive forms include allergic FRS and fungal ball.

Treatment protocol needed for FRS depends upon the time of diagnosis and adequate classification of the disease, which ultimately depends on the demonstration of the fungi. FRS is extremely difficult to treat in certain cases, and it is considered as a major health burden despite multiple advancements in medical and surgical treatments [10-11].

Chronic invasive FRS has two histopathological forms-granulomatous and non-granulomatous-but there is very little difference between the two subtypes. Multiple modalities of treatment are available for chronic FRS, but recurrence after treatment is very common [12-13].

Our finding that male patients were more predominantly affected than females aligns with previous reports that indicate a predilection of chronic FRS for male patients [8,10,14-16]. Also, our age-based findings aligned with previous studies that indicate chronic FRS is more common to patients aged 20 to 40 years [10,14,16]. Most of the patients in our study had a disease history of seven to 12 weeks. The association of FRS to diabetes mellitus was 33.33%, which aligns with the findings by Navya et al. [14] and Driemel et al. [17]. Most patients with chronic FRS were from urban areas with poor socioeconomic status, which aligns with reports by Nazeri et al. [18] and Challa et al. [8]. The frequency of granulomatous invasive FRS in our study is 29.6%. Previous studies by Navya et al. [14] found that 20% of the patients with clinical suspicion of FRS had granulomatous invasive FRS.

Similarly, in a retrospective analysis with 665 cases of rhinosinusitis, 42.7% were of fungal origin; the most common histological subtype was non-invasive allergic FRS, and 16.5% of cases were of chronic invasive granulomatous FRS [19]. Accurate histological diagnosis of the subtype of FRS holds critical value in the approach to treatment. For acute FRS, aggressive surgery and antifungal therapy are initiated. For invasive granulomatous subtypes, surgical removal is supplemented with antifungal treatment. Surgery alone suffices for non-invasive subtypes. Allergic subtypes respond well to corticosteroid monotherapy [19].

This study has greatly emphasized the need for histopathological diagnosis of rhinosinusitis to aid in appropriate management. However, our study was not without limitations. One limitation is that we only included patients with FRS, and therefore, we could not ascertain the incidence of FRS among all other pathologies. This study was limited to a single center, which limits the generalization of the results to other populations. We recommend large scale, multi-center analyses of all rhinosinusitis infections to determine the burden of fungal causes and evaluate the frequencies of its subtypes for future investigations.

## Conclusions

GIFS is highly frequent among patients with clinical suspicion of chronic FRS. Fungal infections should be considered a probable differential diagnosis in patients with chronic rhinosinusitis, even if the patients are immunocompetent. Otolaryngologists should always confirm a histopathological diagnosis of rhinosinusitis before initiating any treatment.

## References

[1] Erskine SE, Verkerk MM, Notley C, Williamson IG, Philpott CM (2016). Chronic rhinosinusitis: patient experiences of primary and secondary care-a qualitative study. Clin Otolaryngol.

[2] Montone KT (2019). Pathology of fungal rhinosinusitis: a review. Head Neck Pathol.

[3] Dykewicz MS, Rodrigues JM, Slavin RG (2018). Allergic fungal rhinosinusitis. J Allerg Clin Immunol.

[4] Cojocari L, Sandul A (2017). Literature review. Noninvasive fungal rhinosinusitis. Roman J Rhinol.

[5] Grosjean P, Weber R (2007). Fungus balls of the paranasal sinuses: a review. Eur Arch Otorhinolaryngol.

[6] Chakrabarti A, Rudramurthy SM, Panda N, Das A, Singh A (2015). Epidemiology of chronic fungal rhinosinusitis in rural India. Mycoses.

[7] Ishaque M, Irshad M, Iqbal M, Dar UF (2015). Outcome of surgical treatment of invasive fungal rhino sinusitis. Pak J Med Health Sci.

[8] Challa S, Uppin SG, Hanumanthu S (2010). Fungal rhinosinusitis: a clinicopathological study from South India. Eur Arch Otorhinolaryngol.

[9] Clark C, Drummond RA (2019). The hidden cost of modern medical interventions: how medical advances have shaped the prevalence of human fungal disease. Pathogens.

[10] Krishnan KU, Agatha D, Selvi R (2015). Fungal rhinosinusitis; a clinicomycological perspective. Indian J Med Micribiol.

[11] Mullings WP, Al-Salman R, Javer AR (2018). Managing allergic fungal rhinosinusitis. Curr Otorhinolaryngol.

[12] Stringer SP, Ryan MW (2000). Chronic invasive fungal rhinosinusitis. Otolaryngol Clin North Am.

[13] Loftus PA, Wise SK (2016). Allergic fungal rhinosinusitis: the latest in diagnosis and management. Adv Otorhinolaryngol.

[14] Navya BN, Vivek TG, Sudhir Sudhir, Kariappa TM, Shwetha VP, Ahalya R (2015). Role of histopathology in the diagnosis of paranasal fungal sinusitis. J Dent Med Sci.

[15] Montone KT, Livolsi VA, Feldman MD (2012). Fungal rhinosinusitis: a retrospective microbiologic and pathologic review of 400 patients at a single university medical center. Int J Otolaryngol.

[16] Karthikeyan P, Coumare VN (2010). Incidence and presentation of fungal sinusitis in patient diagnosed with chronic rhinosinusitis. Indian J Otolaryngol Head Neck Surg.

[17] Driemel O, Wagner C, Hurrass S, Müller-Richter U, Kühnel T, Reichert TE, Kosmehl H (2007). Allergic fungal sinusitis, fungus ball and invasive sinonasal mycosis - three fungal-related diseases [Article in German]. Mund Kiefer Gesichtschir.

[18] Nazeri M, Hashemi SJ, Ardehali M, Rezaei S, Seyedmousavi S, Zareei M, Hosseinjani E (2015). Fungal rhino sinusitisin in Tehran, Iran. Iran J Public Health.

[19] Das A, Bal A, Chakrabarti A, Panda N, Joshi K (2009). Spectrum of fungal rhinosinusitis; histopathologist's perspective. Histopathol.

